# Relationship between Mammographic Density and Age in the United Arab Emirates Population

**DOI:** 10.1155/2019/7351350

**Published:** 2019-08-05

**Authors:** Young-Joon Kang, Soo Kyung Ahn, Seung Ja Kim, Hyeyoung Oh, Jaihong Han, Eunyoung Ko

**Affiliations:** ^1^Department of Surgery, Incheon St. Mary's Hospital, The Catholic University of Korea College of Medicine, Incheon, Republic of Korea; ^2^Department of Surgery, Kangnam Sacred Heart Hospital, Hallym University, Seoul, Republic of Korea; ^3^Department of Radiology, Sheikh Khalifa Specialty Hospital, Ras Al Khaimah, UAE; ^4^Department of Family Medicine, Sheikh Khalifa Specialty Hospital, Ras Al Khaimah, UAE; ^5^Department of Surgery, National Cancer Center, Goyang, Republic of Korea; ^6^Department of Surgical Oncology, Sheikh Khalifa Specialty Hospital, Ras Al Khaimah, UAE

## Abstract

**Objective:**

Higher breast density is a strong, independent risk factor for breast cancer. Breast density varies by age, ethnicity, and geographic area although dense breast tissue has been associated with younger age and premenopausal status. The relationship between breast density and age in women in the United Arab Emirates (UAE) has not been determined. This study evaluated breast density in the UAE population and its relationship with age.

**Methods:**

Women participating in the national cancer screening program from August 2015 to May 2018 who underwent screening mammography were included. Breast parenchymal density was classified according to the American College of Radiology (ACR) Breast Imaging-Reporting and Data System (BI-RADS) from category a (almost entirely fatty) through d (extremely dense). Subjects were divided into six age groups, and the association between age and breast density was evaluated.

**Results:**

Of the 4911 women included, 1604 (32.7%), 2149 (43.8%), 1055 (21.5%), and 103 (2.1%) were classified as having categories a–d breast density, respectively. A significant negative correlation was observed between age and breast density category (*p* < 0.001). Women of mean age 44 ± 7 years had the highest breast density, whereas those of mean age 56 ± 14 years had the lowest breast density. Comparisons of Emirati women with Lebanese and Western women showed that breast density was lower in Emirati women than in the other populations.

**Conclusions:**

To our knowledge, this is the first study to evaluate the relationship between mammographic breast density and age in UAE women. As in other populations, age was inversely related to breast density, but the proportion of Emirati women with dense breasts was lower than in other populations. Because this study lacked demographic, clinical, and histopathological data, further evaluation is required.

## 1. Introduction

Breast cancer is the most common malignancy among women in developed countries and a major cause of cancer deaths in women worldwide. Among women in countries in the Gulf Cooperation Council (United Arab Emirates (UAE), Bahrain, Saudi Arabia, Oman, Qatar, and Kuwait), the incidence of breast cancer was found to have increased 40% over a 12-year period from 1998 to 2009 [1]. Breast cancer is also the most common form of cancer among Emirati women, accounting for 38.8% of all malignancies in 2014, including both citizens (32.16%) and noncitizens (41.41%) of the UAE [2]. Early detection of breast malignancy has been shown to improve patient prognosis and survival [3].

Screening mammography is an effective tool for detecting early breast cancer, thereby reducing rates of morbidity and mortality [4]. Mammography is currently regarded as the only method appropriate for mass screening. However, higher breast density has been strongly associated with decreased mammographic sensitivity [5]. Breast density is measured by determining the ratio of radiodense epithelium and stroma to radiolucent fatty tissue. Higher breast density on mammography is strongly and reproducibly associated with an increased risk of breast cancer, especially in younger women [6–8]. Although postmenopausal changes in glandular breast tissue reduce breast density in an age-dependent manner.

In addition to being related to breast density, age, as well as race, was reported to be associated with changes in mammographic parenchymal patterns [9]. Breast density can vary by ethnic group and geographic area, for reasons that remain unclear [10, 11]. Thus, measurements of breast density are important for risk assessment and prevention strategies. Moreover, knowledge of the ranges of breast density in particular populations is important when planning screening programs.

The Federal Ministry of Health of the UAE initiated a breast cancer screening program in 1995. More recently, the government of the UAE has promoted early cancer detection programs, with the health authorities of Abu Dhabi and Dubai introducing cancer screening programs in 2008 and 2014, respectively. In 2015, the UAE Ministry of Health launched an official cancer screening initiative for breast, cervical, and colorectal cancers. The programs for breast cancer are in accordance with international guidelines, combining monthly breast self-examination, regular clinical breast examination, and mammography biennially beginning at age 40 years [12–16]. To our knowledge, no study to date has evaluated the distribution of mammographic breast density or the relationship between breast density and age in the UAE population. The present study therefore evaluated breast density and its relationship to age among women in the UAE.

## 2. Materials and Methods

Women aged ≥40 years participating in the UAE national cancer screening program who underwent screening mammography from August 2015 to May 2018 were included. And, only citizens participated in the UAE national cancer screening program. Mammograms were performed on FDR 3000AWS (Family Health Promotion Center, SKGH, and DHADNA Health Center), FDR 2000AWS (MUHASNAH Health Center, Fujairah Hospital), Selenia Dimensions (RAK Clinic), Senograph DS ADS (Mother & Child Hospital), Lorad Selenia (VEJTHANI Hospital), and Mammo Diagnost DR (LLH) units. Two mammographic images were obtained from each subject, one in the mediolateral oblique and the other in the craniocaudal plane. All screening mammograms were reviewed retrospectively.

Each mammographic report included an assessment of breast density, as defined by the American College of Radiology (ACR) Breast Imaging-Reporting and Data System (BI-RADS). Breast density was divided into four categories, with category a indicating breasts that were predominantly fatty; b indicating scattered fibroglandular density; c indicating heterogeneously dense breasts, which may obscure small masses; and d indicating extremely dense breast tissue, which reduces the sensitivity of mammography. Breast tissues of categories a and b were considered fatty, and those of categories c and d were considered dense. All mammograms were interpreted by a radiologist with over 10 years of experience in breast imaging. Women were also divided into five age groups (40–49, 50–59, 60–69, 70–79, and ≥80 years), and the association between age group and breast density was analyzed, as was the association between breast density and age as a continuous variable. Correlations between age and breast density were assessed by the ANOVA test. The results in Emirati women were compared with those of Lebanese and Western populations, with statistically significant differences determined by Fisher's exact test. All statistical analyses were performed using SPSS software (version 21, SPSS).

## 3. Results

During the study period, from August 2015 to May 2018, 4911 women underwent mammography. Of these, eight were excluded, three because they were male and five because they lacked BI-RADS density data, with most of the latter having diffuse foreign body granuloma in the breast due to a history of interstitial injection. The remaining 4911 women were considered eligible. Of these 4911 women, 2147 (43.7%), 1727 (35.2%), 803 (16.4%), 198 (4.0%), and 36 (0.7%) were aged 40–49, 50–59, 60–69, 70–79, and ≥80 years, respectively. Mammography results showed that, based on ACR BI-RADS grading, 1604 (32.7%), 2149 (43.8%), 1055 (21.5%), and 103 (2.1%) women were classified into categories a–d, respectively (Table 1).

**Figure 1:**
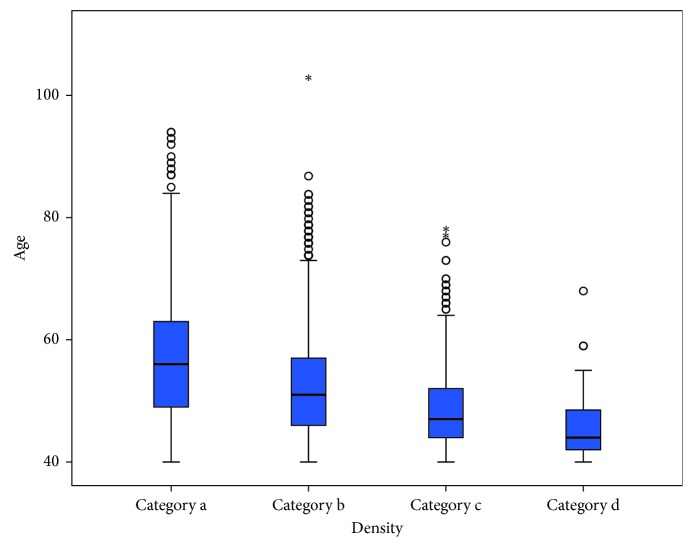
Distribution of breast density classifications by age groups.

Analysis of mammographic breast density by age group showed that breast density was normally distributed (Table 2, Figure 1). Spearman's rank-order correlation analysis showed a significant negative correlation between age and breast density category (*p* < 0.001).

The BI-RADS results for the entire screening cohort are summarized in Table 3. Of the 4911 subjects, 4655 (94.8%) had normal or benign screening results, whereas 16 (0.3%) were classified into BI-RADS categories 4 and 5 (Table 3).

**Figure 2:**
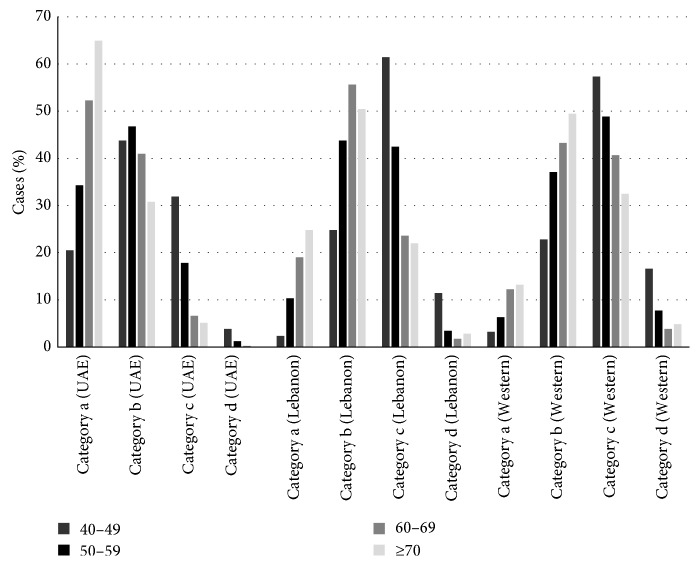
Bar graph showing patients' age and categories of breast density by researches.

**Figure 3:**
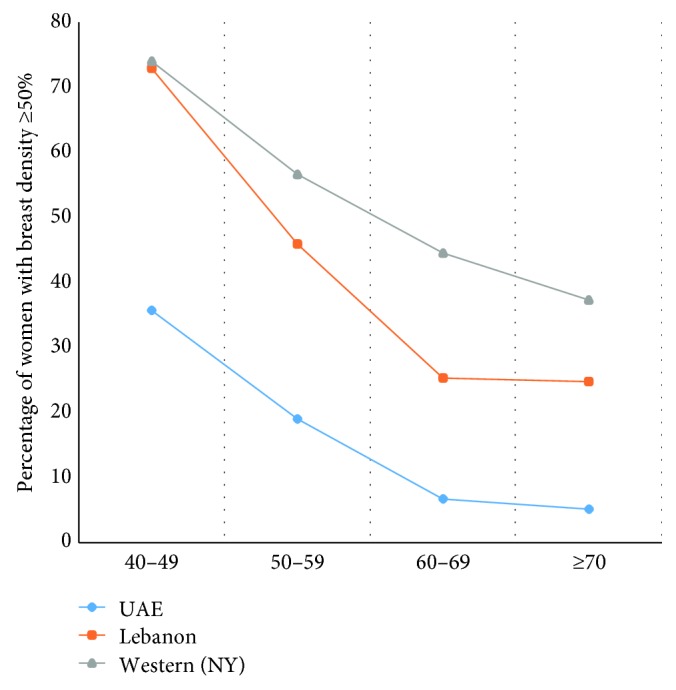
Comparison in the percentage of females with breast density of 50% and above between our study's results and the studies performed in Lebanon [17] and New York [18].

These results in Emirati women were compared with published results in Lebanese and Western women [17, 18] (Table 4, Figure 2). These studies also found a negative correlation between breast density and age. However, the percentage of Emirati women with lower breast density was higher than the percentages of Lebanese and Western women over all ages (Figure 3).

## 4. Discussion

Breast cancer is a major health concern among Emirati women. In the UAE, cancer is the third leading cause of death [19] and breast cancer is the most frequent type of cancer in women [2]. Nevertheless, only 10% of Emirati women undergo breast screening, and over 65% of breast malignancies are diagnosed in advanced stages [20]. It is therefore important to raise awareness and introduce regular screening methods for breast cancer in the UAE.

In many developed countries, breast cancer screening includes mammography performed every 2 years. This screening method has reduced breast cancer mortality rates, especially among women aged 50–69 years, and it is recommended by the European Union and numerous individual countries [21]. Screening mammography is effective in Western women aged >40 years. Its sensitivity and specificity are age-dependent, being higher in older women [22].

Earlier screening has been recommended in Arab populations [23]. Emirati women tend to develop breast cancer at least 10 years earlier than women in Western countries [2, 23, 24]. For example, 25.5% of breast cancers develop in women aged <40 years living in Gulf Cooperation Council countries, compared with <5% in women aged <40 years living in Western countries [1]. The clinicopathological and immunohistochemical features of breast cancer differ in Arab and Western populations [24, 25]. Younger women tend to have denser breast tissue, with higher ratios of glandular tissue to fat, than older women. This study showed that breast density on mammography was generally lower in Emirati than in Western women over all age groups. The results of screening mammography in the UAE should be adjusted according to local data rather than to international and foreign data. Based on the results of this study, which showed that breast density in Emirati women is lower than in general occidental and Lebanese population, breast density is not by itself or alone responsible for early age breast cancer incidence in the Emirates. Changing in screening mammography programs should take into consideration other factors than breast density in the Emirates to explain early onset incidence of breast cancer. Therefore, nowadays, no change in breast cancer screening program is advisable.

This study had several limitations. First, this study did not include demographic data on the study population, such as history of breast feeding and hormonal replacement therapy, body mass index, menopausal status, parity, and personal and familial history of breast cancer. There were also no clinico-histopathological data. Determining the incidence of breast cancer is required to evaluate the relationship between mammographic breast density and breast cancer risk factors in Emirati women. These sources were difficult to collect the personal data because these were a UAE national cancer screening program. Additional data are required to improve access to breast cancer screening. Therefore, data will be collected and analyzed for follow-up women in hospitals where it is possible to collect data among Emirati women enrolled in the screening program. In addition, the interpretation of mammographic density category may vary among radiologists [26]. The reproducibility of BI-RADS breast density among radiologists may be improved by continued monitoring of their density classifications, with variations among radiologists reduced by the implementation of educational and/or quality assurance measures [26].

Barriers to breast cancer screening have been found to include costs, time concerns, quality of care issues, fear, and medical concerns overlapping with clinical and service quality initiatives. Health system-related barriers have also been identified, including access to insurance, physician recommendation, physician gender, provider characteristics, having a regular provider, fear of the system or procedure, and knowledge of the health system, all of which can be addressed within the UAE [27].

## 5. Conclusions

To our knowledge, this study is the first to evaluate the relationship between mammographic breast density and age in Emirati women. As in other populations, age was inversely related to breast density, but the proportion of Emirati women with dense breasts was lower than in other populations. The present study indicates that regular screening mammography is effective among women in the UAE.

Although many studies, in various regions of the world, have assessed the epidemiology of breast cancer, few have assessed the incidence of breast cancer among women in the UAE. This paper has significance to provide the breast screening basic data and to determine the relationship between breast density and age in the Emirati women. Our findings indicate the importance of tracking demographic and cancer information in Emirati and Arab women, and the use of that information to establish local screening and diagnostic and therapeutic guidelines.

## Figures and Tables

**Table 1:** ACR type results from screening mammography.

ACR BI-RADS density	Number of women	Minimum age in years	Maximum age in years	5% trimmed mean age in years	SD in years
a	1604	40	94	55.9	9.7
b	2149	40	103	52.1	8.3
c	1055	40	78	48.4	6.3
d	103	40	68	45.6	4.8
Total	4911	40	103	52.6	8.9

ACR category a: predominantly fatty (low density); category b: scattered fibroglandular densities (average density); category c: heterogeneously dense (high density); category d: extremely dense (very high density).

**Table 2:** Distribution of mammographic density with age group.

Age group (years)	ACR category a	ACR category b	ACR category c	ACR category d	Total
40∼49	440 (20.5%)	940 (43.8%)	685 (31.9%)	82 (3.8%)	2147
50∼59	592 (34.3%)	808 (46.8%)	307 (17.8%)	20 (1.2%)	1727
60∼69	420 (52.3%)	329 (41.0%)	53 (6.6%)	1 (0.1%)	803
70∼79	127 (64.1%)	61 (30.8%)	10 (5.1%)	0 (0.0%)	198
≥80	25 (69.4%)	11 (30.6%)	0 (0.0%)	0 (0.0%)	36
Total	1604 (32.7%)	2149 (43.8%)	1055 (21.5%)	103 (2.1%)	4911

**Table 3:** BI-RADS results from screening mammography of 4911 patients in the study group.

BI-RADS category	No. (%) of patients	Recommendation
0	240 (4.9)	Recall; additional imaging
1	3448 (70.2)	Routine biennial mammography
2	1185 (24.1)	Routine biennial mammography
3	22 (0.4)	Short-interval follow-up; repeat mammography in 6 months
4	8 (0.2)	Suspicious abnormality; biopsy recommended
5	8 (0.2)	Highly suggestive of malignancy; biopsy recommended

**Table 4:** Distribution of breast density according to age of UAE, Lebanon, and New York women.

ACR category	Country	Age group (%)			
		40–49	50–59	60–69	≥70
a	UAE	440 (20.5)	592 (34.3)	420 (52.3)	152 (65.0)
	Lebanon	8 (2.3)	30 (10.3)	33 (19.0)	27 (24.8)
	Western (NY)	53 (3.2)	137 (6.3)	200 (12.2)	165 (13.2)

b	UAE	940 (43.8)	808 (46.8)	329 (41.0)	72 (30.8)
	Lebanon	87 (24.8)	128 (43.8)	97 (55.7)	55 (50.5)
	Western (NY)	382 (22.8)	814 (37.1)	710 (43.3)	620 (49.5)

c	UAE	685 (31.9)	307 (17.8)	53 (6.6)	10 (5.1)
	Lebanon	216 (61.5)	124 (42.5)	41 (23.6)	24 (22.0)
	Western (NY)	962 (57.4)	1072 (48.9)	667 (40.7)	407 (32.5)

d	UAE	82 (3.8)	20 (1.2)	1 (0.1)	0 (0.0)
	Lebanon	40 (11.4)	10 (3.4)	3 (1.7)	3 (2.8)
	Western (NY)	278 (16.6)	169 (7.7)	62 (3.8)	60 (4.8)

Total	UAE	2147	1727	803	234
	Lebanon	351	292	174	109
	Western (NY)	1675	2192	1639	1252

## Data Availability

The demographic and clinical data collected for the purpose of the statistical analysis to support the findings of this study are available from the corresponding author upon request.

## References

[1] Al-Madouj A. N., Al-Zahrani A. S., Al-Othman S. F. R. (2013). *Cancer Incidence Among Nationals of the GCC States, 1998–2009*.

[2] Cancer Incidence in United Arab Emirates (2014). Annual report of the UAE-national cancer registry.

[3] Tabár L., Vitak B., Chen H.-H. (2000). The Swedish two-county trial twenty years later. *Radiologic Clinics of North America*.

[4] Humphrey L. L., Helfand M., Chan B. K. S., Woolf S. H. (2002). Breast cancer screening: a summary of the evidence for the U.S. preventive services task force. *Annals of Internal Medicine*.

[5] Foulkes W. D., Reis-Filho J. S., Narod S. A. (2010). Tumor size and survival in breast cancer—a reappraisal. *Nature Reviews Clinical Oncology*.

[6] Vachon C. M., van Gils C. H., Sellers T. A. (2007). Mammographic density, breast cancer risk and risk prediction. *Breast Cancer Research*.

[7] Pinsky R. W., Helvie M. A. (2010). Mammographic breast density: effect on imaging and breast cancer risk. *Journal of the National Comprehensive Cancer Network*.

[8] Boyd N. F., Guo H., Martin L. J. (2007). Mammographic density and the risk and detection of breast cancer. *New England Journal of Medicine*.

[9] Zulfiqar M., Rohazly I., Rahmah M. (2011). Do the majority of Malaysian women have dense breasts on mammogram?. *Biomedical Imaging and Intervention Journal*.

[10] Boyd N. F., Martin L. J., Rommens J. M. (2009). Mammographic density: a heritable risk factor for breast cancer. *Methods in Molecular Biology*.

[11] Vachon C. M., Sellers T. A., Carlson E. E. (2007). Strong evidence of a genetic determinant for mammographic density, a major risk factor for breast cancer. *Cancer Research*.

[12] Pearlman D. N., Clark M. A., Rakowski W., Ehrich B. (1999). Screening for breast and cervical cancers: the importance of knowledge and perceived cancer survivability. *Women & Health*.

[13] Sadler G. R., Dhanjal S. K., Shah N. B. (2001). Asian Indian women: knowledge, attitudes and behaviors toward breast cancer early detection. *Public Health Nursing*.

[14] HAAD Cancer Programs, 2018

[15] Breast Cancer Screening Available at DHA Centres, 2018

[16] Ministry of Health Launches (2018). *The National Periodic Health Screening and Cancer Screening Initiatives*.

[17] Salem C., Atallah D., Safi J. (2017). Breast density and breast cancer incidence in the Lebanese population: results from a retrospective multicenter study. *BioMed Research International*.

[18] Checka C. M., Chun J. E., Schnabel F. R., Lee J., Toth H. (2012). The relationship of mammographic density and age: implications for breast cancer screening. *American Journal of Roentgenology*.

[19] Loney T., Aw T.-C., Handysides D. G. (2013). An analysis of the health status of the United Arab Emirates: the Big 4 public health issues. *Global Health Action*.

[20] Taher J A. N. G., Sabih W. Community profile summary of findings. http://www.haad.ae.

[21] Association of European Cancer Leagues (2017). *European Union Council Recommendation on Cancer Screening*.

[22] Armstrong K., Moye E., Williams S., Berlin J. A., Reynolds E. E. (2007). Screening mammography in women 40 to 49 years of age: a systematic review for the American College of Physicians. *Annals of Internal Medicine*.

[23] Najjar H., Easson A. (2010). Age at diagnosis of breast cancer in Arab nations. *International Journal of Surgery*.

[24] Chouchane L., Boussen H., Sastry K. S. R. (2013). Breast cancer in Arab populations: molecular characteristics and disease management implications. *The Lancet Oncology*.

[25] Al Tamimi D. M., Shawarby M. A., Ahmed A., Hassan A. K., AlOdaini A. A. (2010). Protein expression profile and prevalence pattern of the molecular classes of breast cancer—a Saudi population based study. *BMC Cancer*.

[26] Spayne M. C., Gard C. C., Skelly J., Miglioretti D. L., Vacek P. M., Geller B. M. (2012). Reproducibility of BI-RADS breast density measures among community radiologists: a prospective cohort study. *The Breast Journal*.

[27] Bowser D., Marqusee H., El Koussa M., Atun R. (2017). Health system barriers and enablers to early access to breast cancer screening, detection, and diagnosis: a global analysis applied to the MENA region. *Public Health*.

